# Melioidosis in Children, Brazil, 1989–2019

**DOI:** 10.3201/eid2805.211473

**Published:** 2022-05

**Authors:** Bijayini Behera, Anjuna Radhakrishnan, Sonali Mohapatra, Baijayantimala Mishra

**Affiliations:** All India Institute of Medical Sciences, Bhubaneswar, India

**Keywords:** melioidosis, *Burkholderia pseudomallei*, bacteria, pediatric, Brazil, India

**To the Editor:** We read with great interest the article by Lima et al. (*1*), in which the authors have discussed 20 confirmed or suspected melioidosis cases in children over a period of 30 years, concluding that childhood melioidosis is more severe in Brazil. This conclusion seems far-fetched based on findings described in the article, although the authors state that the high death rate and clinical severity might have been attributed to underreporting of mild cases,

Melioidosis is not a notifiable disease in India. Even so, from a single tertiary-care teaching hospital at Odisha, we have reported >100 cases of culture-confirmed cases during 2016–2021 (*2*–*4*), of which 10 cases were in the pediatric population (8 cases of superficial pyogenic infections in otherwise healthy children and 2 cases of septicemic melioidosis). The second septicemic case was a 3-year-old girl with underlying acute lymphoblastic leukemia; she was treated with intravenous meropenem for 10 days and was discharged with a regimen of oral cotrimoxazole for 12 weeks.

Clinical severity of melioidosis is predominantly a function of host immunity (*5*). At a more pragmatic level, we would like to emphasize that, in melioidosis-endemic regions, most immunocompetent children with melioidosis experience localized infections and have better clinical outcomes, whereas in children with risk factors such as immunosuppression and childhood malignancies, the clinical course may be sudden and severe. In our view, frequent environmental exposures may not entirely explain the severity of childhood melioidosis. Lima et al. should have provided additional evidence to support their conclusion that childhood melioidosis is more severe in the population in Brazil.
